# Group B Streptococcal Hemolytic Pigment Impairs Platelet Function in a Two-Step Process

**DOI:** 10.3390/cells11101637

**Published:** 2022-05-13

**Authors:** Kristin Jahn, Patience Shumba, Phoenicia Quach, Mathias Müsken, Jan Wesche, Andreas Greinacher, Lakshmi Rajagopal, Sven Hammerschmidt, Nikolai Siemens

**Affiliations:** 1 Center for Functional Genomics of Microbes, Department of Molecular Genetics and Infection Biology, Interfaculty Institute for Genetics and Functional Genomics, University of Greifswald, 17489 Greifswald, Germany; kristin.jahn@uni-greifswald.de (K.J.); patience.shumba@uni-greifswald.de (P.S.); 2 Department of Global Health, University of Washington, Seattle, WA 98105, USA; pjfquach@gmail.com (P.Q.); lakshmi.rajagopal@seattlechildrens.org (L.R.); 3 Central Facility for Microscopy, Helmholtz Centre for Infection Research, 38124 Braunschweig, Germany; mathias.muesken@helmholtz-hzi.de; 4 Department of Transfusion Medicine, Institute of Immunology and Transfusion Medicine, University Medicine Greifswald, 17475 Greifswald, Germany; wesche@uni-greifswald.de (J.W.); andreas.greinacher@med.uni-greifswald.de (A.G.); 5 Center for Global Infectious Disease Research, Seattle Children’s Research Institute, Seattle, WA 98019, USA; 6 Department of Pediatrics, University of Washington School of Medicine, Seattle, WA 98105, USA

**Keywords:** *Streptococcus agalactiae*, group B streptococcus, pigment, platelets

## Abstract

Group B streptococci (GBS) cause a range of invasive maternal–fetal diseases during pregnancy and post-partum. However, invasive infections in non-pregnant adults are constantly increasing. These include sepsis and streptococcal toxic shock syndrome, which are often complicated by systemic coagulation and thrombocytopenia. GBS express a hyper-hemolytic ornithine rhamnolipid pigment toxin with cytolytic and coagulatory activity. Here, we investigated the effects of GBS pigment on human platelets. Infections of platelets with pigmented GBS resulted initially in platelet activation, followed by necrotic cell death. Thus, this study shows that GBS pigment kills human platelets.

## 1. Introduction

*Streptococcus agalactiae*, or group B streptococci (GBS), are a major cause of morbidity and mortality in neonates and pregnant women. Since the 1980s, reports of invasive infection in non-pregnant adults with underlying conditions have increased [1]. These include necrotizing skin and soft tissue infections (NSTIs), which are often accompanied by sepsis or streptococcal toxic shock syndrome (STSS) [1]. Sepsis, particularly STSS, is associated with excessive hyper-inflammation, leading to multi-organ failure [2]. Common additional complications include disseminated intravascular coagulation (DIC) and thrombocytopenia [3], which are associated with poor prognosis [4].

Platelets are the second most abundant cell type in the circulation. Despite their role in hemostasis, they also function as immune cells. Upon activation, platelets release their granule contents, comprising of bactericidal proteins, cytokines, coagulation factors, and immunomodulatory factors [4]. Researchers have reported that several bacterial species interact with platelets, resulting in platelet activation or damage [4]. So far, platelet activation has predominantly been reported by GBS strains isolated from septic patients [5]. Recently, an unusual case involving STSS, DIC, and *purpura fulminans* in an immunocompetent individual was reported [6]. The causative GBS strain was pigmented and hyper-hemolytic. The GBS pigment, which is a surface localized ornithine rhamnolipid also called Granadaene [7], showed pro-inflammatory and coagulatory activity [6]. Here, we analyzed the impact of GBS pigment on human platelet function.

## 2. Materials and Methods

### 2.1. Ethics

The use of whole blood and washed platelets from healthy adults was approved by the Ethics Committee of the University Medicine Greifswald (BB 044/18). All volunteers gave written informed consent in accordance with the Declaration of Helsinki. All experiments were carried out in accordance with the approved guidelines.

### 2.2. Platelet Preparation

Washed human platelets were prepared as described previously [8]. In brief, platelet rich plasma (PRP) was prepared from ACD-A anticoagulated whole blood from a defined set of healthy volunteers who were not taking any anti-platelet or non-steroidal anti-inflammatory drugs. The PRP was washed two times in Tyrode’s buffer containing 2.5 U/mL apyrase, 1 U/mL hirudin, 0.35% BSA, and 0.1% glucose, with a pH of 6.3. Lastly, the platelet pellet was resuspended in a bicarbonate-based suspension buffer supplemented with 2.12 mM MgCl_2_, 1.96 mM CaCl_2_, 0.35% BSA, and 0.1% glucose, with a pH of 7.2. The platelet count was adjusted to 300,000 platelets/µL.

### 2.3. Bacterial Strains and Pigment Purification

The following GBS strains isolated from NSTI and/or STSS patients were used: pigmented LUMC16 and STSS/NF-HH, and non-pigmented STSS/NF-LH [6,9]. The LUMC16Δ*cylX-K* mutant was constructed using methods described previously [7]. Briefly, the plasmid pHY304Δ*cylX-K* was electroporated into LUMC16, and selection and screening for the double crossover mutant was performed. PCR was used to verify the absence of *cylX-K* and the presence of Ω *km*-2, using primers for flanking genomic regions and internal gene primers as described previously [7]. The colonies were examined for the loss of hemolysis and pigmentation on blood agar and Granada media, respectively. All strains were cultured in Todd–Hewitt broth supplemented with 1.5% (*w*/*v*) yeast extract (Roth).

The GBS pigment from the STSS/NF-HH strain was purified as previously described [6]. Parallel extraction from the non-pigmented STSS/NF-LH strain was performed and used as a control. To confirm that the pigment was extracted, and for quantification, MALDI-FTICR-MS was performed [6,7]. The pigment was exclusively present in the samples from the STSS/NF-HH strain (*m*/*z* value of 677.3862), whereas no peak was detected in the extracts from the STSS/NF-LH strain. This extract was used in all experiments as a negative buffer control. A hemoglobin release assay was performed to confirm the hemolytic activity of the pigment [6].

### 2.4. Platelet Infections

All experiments were performed in Tyrode’s buffer containing Ca^2+^ and Mg^2+^. The platelets were infected with increasing multiplicity of infection (MOI) for LUMC16, LUMC16Δ*cylX-K* (MOI 0.1, MOI 1.0, MOI 10), STSS/NF-HH, and STSS/NF-LH (MOI 0.1), or were incubated with increasing concentrations of isolated pigment or the respective buffer control (0.5, 1.0, 2.0 µM) for 30, 60, and 120 min. The platelet releasate was prepared by the activation of 2.1 × 10^7^ platelets with 40 µM TRAP-6 and 30 µg/mL collagen for 10 min at 37 °C. The activated platelets were pelleted, and supernatants were used as a releasate. Washed platelets or platelet releasate were incubated with 2.1 × 10^6^ CFU (MOI 0.1). The CFUs were determined by plating serial dilutions on blood agar plates.

### 2.5. Platelet Activation Assay and Assessment of Death

Activation assays were performed in Tyrode’s buffer containing Ca^2+^ and Mg^2+^ as described previously [8]. The washed human platelets were infected as described above. The CD62P expression of CD42a-positive cells was measured using a FACSCalibur (Becton Dickinson, Franklin Lakes, NJ, USA) flow cytometer and CellQuestPro 6.0, and was analyzed using FCS Express 7 Software (De Novo Software). The gating strategy is displayed in Figure S1A. In a subset of experiments, platelets were subsequently stimulated with 20 µM TRAP-6 5 min prior to the end of stimulations/infections. The following antibodies were used: PE-Cy5-labelled monoclonal mouse anti-human CD62P and FITC-labelled mouse anti-human CD42a (BD Biosciences, Franklin Lakes, NJ, USA).

Platelet viability was determined using a RealTime-Glo Cell Viability Assay kit (Promega, Walldorf, Germany) over a period of 120 min. The washed human platelets were infected with GBS or were stimulated with the pigment, as described above. One minute after mixing and shaking the plate (300 rpm; 3 s), luminescence was recorded every 180 s. PBS and TritonX-100 (Sigma-Aldrich, Taufkirchen, Germany) served as negative and positive controls, respectively. All experiments were performed in duplicates.

A FAM-FLICA caspase-3/7 assay kit (ImmunoChemistry, Davis, CA, USA), TMRE-mitochondrial membrane potential assay kit (Abcam), and Annexin V-FITC (BioLegend) were used to determine apoptotic and/or necrotic events after 120 min of infection or stimulation. All experiments were performed according to the manufacturer’s instructions. The PBS and ABT-737 (10 µM, Hycultec, Beutelsbach, Germany), FCCP (20 µM), and Ply (300 ng/ml) stimulations served as negative or positive controls. All measurements were performed using a FACSCalibur (Becton Dickinson, Franklin Lakes, NJ, USA) flow cytometer and CellQuestPro 6.0.

### 2.6. Field Emission Scanning Electron Microscopy

Field emission scanning electron microscopy (FESEM) was performed as previously described [8]. Briefly, the infected platelets were fixed with 5% formaldehyde and 2% glutaraldehyde in 0.1 M Hepes buffer (0.1 M HEPES, 0.09 M sucrose, 10 mM CaCl_2_, 10 mM MgCl_2_, pH 6.9). The samples were centrifuged at 2000× *g* for 2 min, washed with TE buffer (20 mM Tris-HCl, 2 mM EDTA, pH 6.9), and resuspended in 50 µL of TE buffer. The cells were placed onto poly-l-lysine-coated cover slips, fixed with 1% glutaraldehyde in TE buffer for 10 min, washed with TE buffer, dehydrated with an increasing series of acetone, underwent critical point drying (CPD 300; Leica, Wetzlar, Germany), and were sputter-coated with gold–palladium (SCD 500; Bal-Tec, Los Angeles, CA, USA). For imaging via a field emission scanning electron microscope (Zeiss Merlin), Everhart–Thornley and In-lens SE detectors (at a ratio of 25:75 and an acceleration voltage of 5 kV) and SmartSEM software 6.06 were used.

### 2.7. Statistics

If not otherwise indicated, statistically significant differences were determined using Kruskal Wallis tests with Dunn’s post-tests. Statistical analyses were performed using GraphPad Prism version 7 (GraphPad software). A *p*-value less than 0.05 was considered significant.

## 3. Results

### 3.1. Pigmented Hyper-Hemolytic GBS Strains Induce Initial Platelet Activation

**Figure 1:**
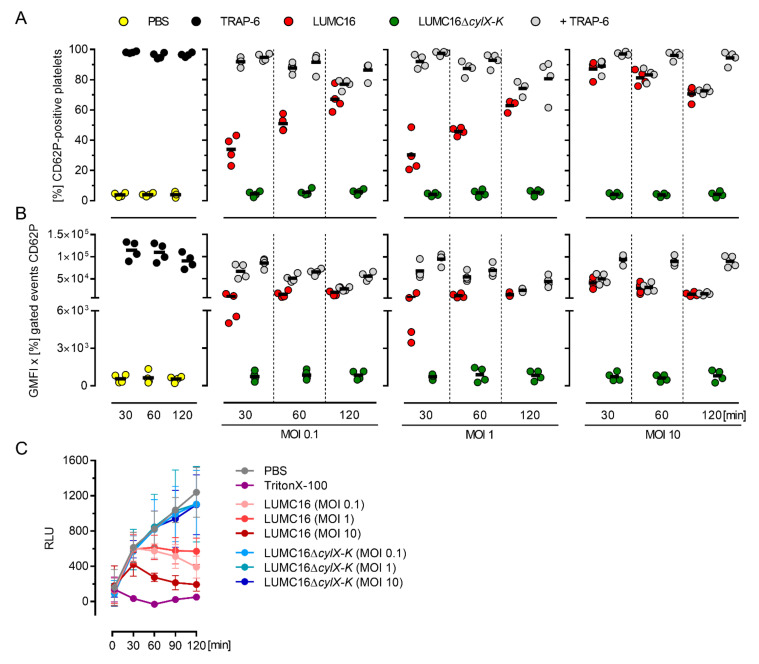
Pigmented GBS activate and kill human platelets. Washed human platelets were infected with the pigmented LUMC16 and the non-pigmented LUMC16Δ*cylX-K* GBS strains at MOI 0.1, MOI 1.0, and MOI 10. (A) Platelet activation was measured via flow cytometry using a PE-Cy5 labelled CD62P antibody. TRAP-6 (40 µM) and PBS were used as positive and negative controls, respectively. Alternatively, 5 min prior to the end of infection, TRAP-6 (40 µM) was added. The activation process was evaluated by assessing frequencies of CD62P-positive cells (**A**) as well as expression of CD62P (**B**). Each dot in A and B represents one independent experiment with washed platelets from one donor (*n* = 4). Horizontal lines depict mean values. (**C**) Kinetics of platelet viability. PBS and TritonX-100 were used as controls. Each dot represents the mean ± SD from four independent experiments (*n* = 4). Abbreviations (GMFI, geometric mean of fluorescence intensity; RLU, relative luminescence units).

To assess the impact of GBS pigment on platelet activation, the pigmented LUMC16 strain and its non-pigmented isogenic LUMC16Δ*cylX-K* mutant were used. Platelets were exposed to bacteria, and their activation was determined via CD62P-positive surface staining. Irrespective of the MOI, the platelets responded to stimulations with the LUMC16 strain within 30 min (Figure 1A,B and Figure S1B). However, no further activation was evident at later time points (60 and 120 min). To determine whether additional activation could be achieved, TRAP-6 (a thrombin receptor agonist) was added to the infections five minutes prior to termination of the experiment. Both the frequencies of CD62P-positive cells and CD62P expression increased after 30 and 60 min of infection when low MOIs (0.1 and 1.0) were used. The platelets remained unresponsive to TRAP-6 stimulation at a high MOI of 10. In contrast, we did not observe a response to infections with the non-pigmented LUMC16Δ*cylX-K* strain (Figure 1A,B and Figure S1B).

**Figure 2:**
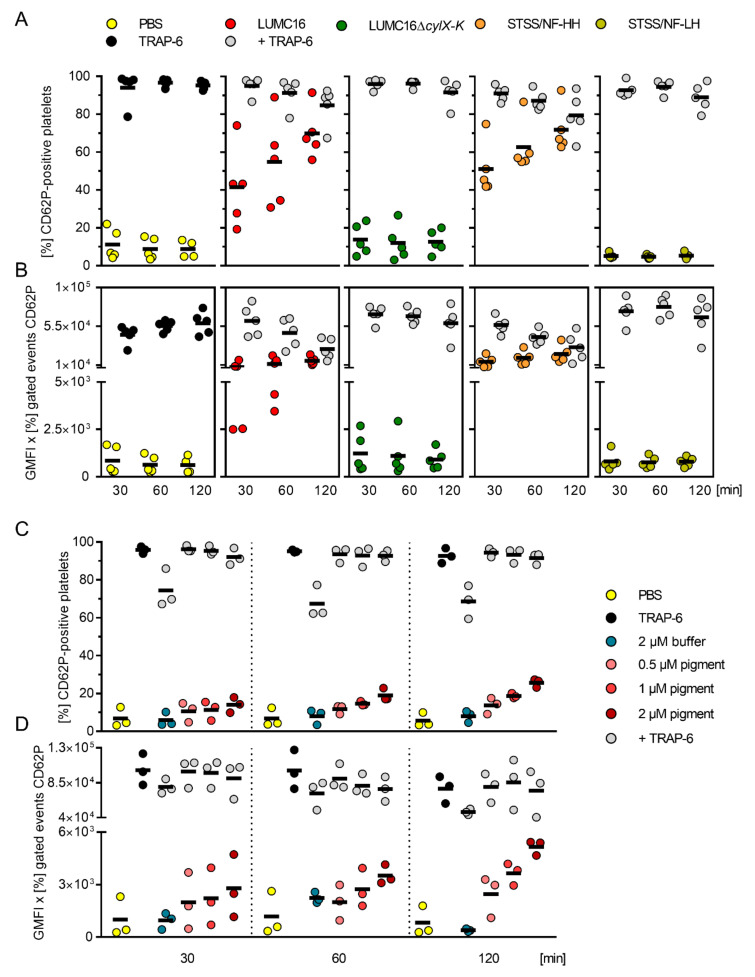
GBS pigment initially activates human platelets. Washed human platelets were infected with pigmented (LUMC16 and STSS/NF-HH) and non-pigmented (LUMC16Δ*cylX-K* or STSS/NF-LH) GBS strains at MOI 0.1 or were incubated with increasing concentrations of the pigment. Platelet activation was measured via flow cytometry using a PE-Cy5 labelled CD62P antibody. TRAP-6 (40 µM) and PBS were used as positive and negative controls, respectively. Alternatively, 5 min prior to the end of infection or pigment stimulation, TRAP-6 was added. The activation process was evaluated by assessing frequencies of CD62P-positive cells (**A**,**C**) as well as expression of CD62P (**B**,**D**). Each dot represents one independent experiment with cells from one donor (A and B, *n* = 5; C and D, *n* = 3). Horizontal lines depict mean values. Abbreviations (GMFI, geometric mean of fluorescence intensity).

In order to verify that activation was not only linked to the DIC/STSS case strain, two previously characterized GBS phenotypic variants, pigmented hyper-hemolytic and non-pigmented low-hemolytic variants (HH and LH, respectively) isolated from the same tissue culture of an STSS/NF case, were included [6,9]. All infections were performed with an MOI of 0.1. Again, both pigmented strains activated platelets within 30 min, and the cells maintained their responsiveness to the additional TRAP-6 stimulations for another 30 min (Figure 2A,B and Figure S1C). In contrast, the platelets remained unaffected in infections with non-pigmented strains, and responded exclusively to additive TRAP-6 stimulations (Figure 2A,B).

Next, pigment extracts from the STSS/NF-HH and STSS/NF-LH (buffer control) strains were used. The dose- and time-dependent activation of platelets was observed in response to hemolytic pigment (Figure 2C,D and Figure S2A). A subpopulation of pigment-stimulated platelets responded to the additional TRAP-6 stimulation. In contrast, no response to extracts from the STSS/NF-LH strain was noted (Figure 2C,D and Figure S2A; “buffer”).

### 3.2. Hemolytic GBS Pigment Causes Platelet Death

**Figure 3:**
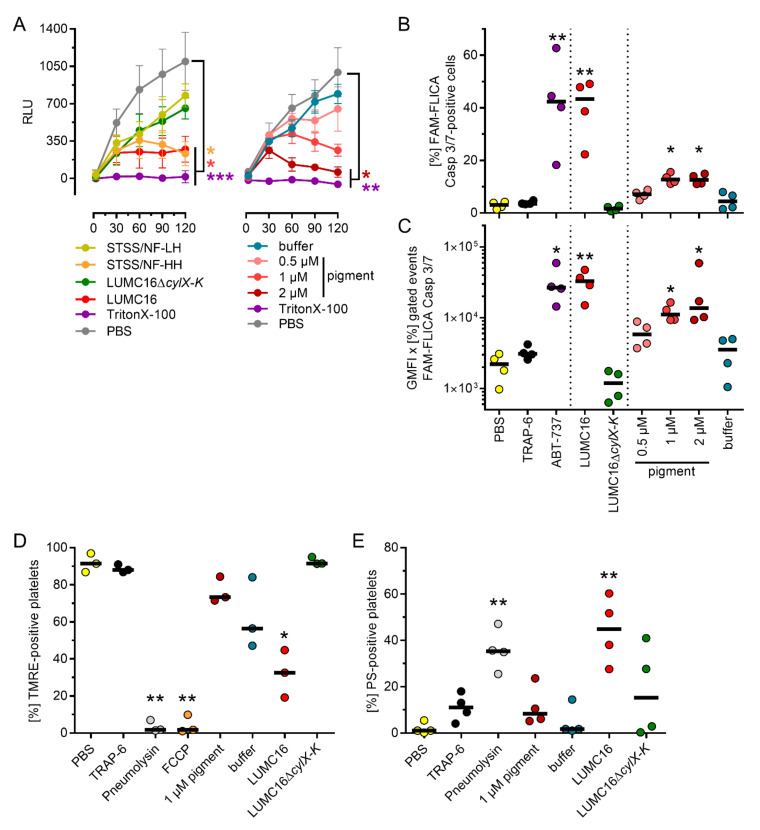
GBS pigment kills platelets. (**A**) Kinetics of platelet viability. PBS and TritonX-100 were used as negative and positive controls, respectively. (**B**,**C**) Caspase-3/7 activity in GBS-infected or pigment-treated platelets after two hours of exposure. The process was evaluated by assessing frequencies of Caspase 3/7-positive cells (**B**) as well as expression of Caspase 3/7 (**C**). PBS and ABT-737 (10 µM) were used as negative and positive controls, respectively. (**D**) Frequencies of TMRE-positive platelets after two hours of exposure to bacteria or pigment. Pneumolysin (300 ng/mL) and FCCP (20 µM) were used as controls. (**E**) Frequencies of PS-positive platelets after two hours of exposure to bacteria or pigment. Pneumolysin (300 ng/mL) was used as a control. Each dot in A represents the mean ± SD from four independent experiments (*n* = 4). Each dot in B-E represents one independent experiment with cells from one donor (B and C, *n* = 4; D, *n* = 3; E, *n* = 4). Horizontal lines depict mean values. The level of significance was determined using Kruskal-Wallis tests with Dunn’s post-tests (*, *p* < 0.05; **, *p* < 0.01; ***, *p* < 0.001). Abbreviations (GMFI, geometric mean of fluorescence intensity; RLU, relative luminescence units; TMRE, tetramethylrhodamine ethyl ester; FCCP, carbonyl cyanide 4-(trifluoromethoxy) phenylhydrazone; PS, phosphatidylserine).

To assess the reason for limited platelet activation at later stages of infection, platelet viability was determined. The infection dose- and time-dependent killing of platelets by the pigmented GBS was observed (Figure 1C and Figure 3A). In contrast, the cells remained viable in infections with non-pigmented strains (Figure 1C and Figure 2A). Consequently, the platelets were stimulated with increasing concentrations of the pigment. The platelets were viable only for 30 and 60 min when high concentrations (1 and 2 µM) of the pigment were used (Figure 3A).

The TRAP-6 unresponsiveness in activation assays, as well as the limited substrate utilization in viability experiments, showed that the pigment kills platelets at later stages of infection/stimulation. This is also supported by the increased PS-positivity of the cells, particularly in response to the pigmented LUMC16 strain (Figure 3 and Figure S2). To determine the type of cell death, platelets were exposed to bacteria or pigment for 120 min and caspase-3/7 activity was determined. The LUMC16 strain, as well as higher concentrations of the pigment (1 and 2 µM), induced caspase-3/7 activity in the platelets (Figure 3B,C). Only baseline levels of caspase-3/7 were detected in stimulation/infection when the pigment was not present. To differentiate between apoptotic and necrotic cell death, the inner mitochondrial membrane potential was measured via TMRE labeling of the mitochondria after 120 min of stimulation/infection. The stimulation of platelets with 1 µM pigment led to a slight decrease in mitochondrial membrane potential. In contrast, the infection of platelets with the LUMC16 strain resulted in diminished membrane potential. This was comparable to the FCCP control. Stimulation of platelets with the buffer control also resulted in reduced mitochondrial membrane potential without any evidence of platelet killing (Figure 3C and Figure S2). Infection with the non-pigmented LUMC16Δ*cylX-K* did not alter mitochondrial membrane potential.

**Figure 4:**
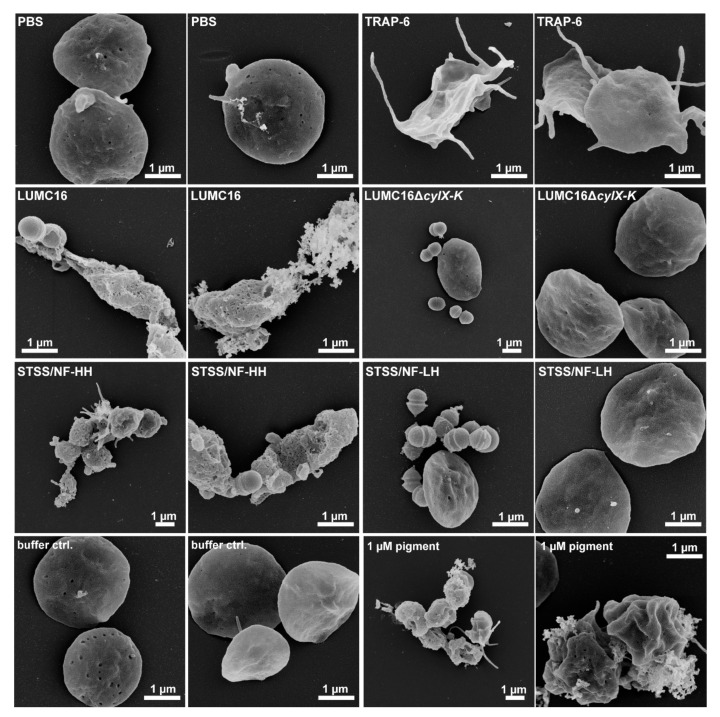
Platelet morphology in response to GBS infection or pigment stimulation. Washed human platelets were infected with indicated GBS strains or stimulated with pigment (1 µM) for 120 min and were visualized via FESEM. PBS and TRAP-6 were used as negative and activation controls, respectively. Representative images from three independent experiments are shown (*n* = 3).

The phenotypic assessment of platelets via electron microscopy confirmed the observations described above. The platelets showed a resting-like phenotype in infections with non-pigmented strains. In contrast, the platelets that were exposed to the pigmented strains or the pigment itself were characterized by ruptured membranes and surface-bound granule content (Figure 4).

**Figure 5:**
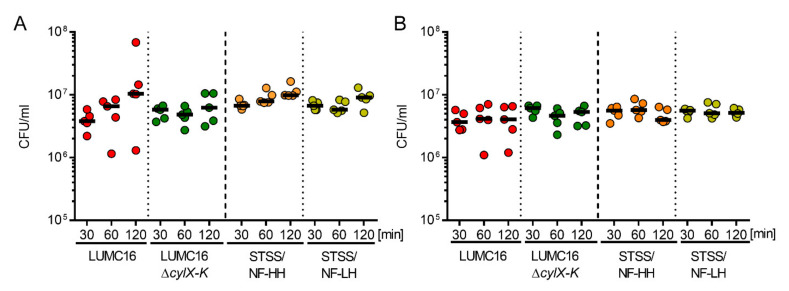
Human platelets do not kill GBS. CFU of the indicated GBS strains after 30, 60, and 120 min of platelet infection (**A**) or incubation with platelet releasate (**B**). Each dot represents one independent experiment with washed platelets from one donor (*n* = 5). Horizontal lines depict mean values. Abbreviations (CFU, colony-forming units).

Although platelets (especially platelet releasate) exert antimicrobial activity (e.g., against *S. aureus* [10]), dividing bacteria were detected via electron microscopy (Figure 4). Therefore, bacterial survival was assessed during the entire period of platelet or releasate exposure. All GBS strains survived regardless of the pigmentation (Figure 5).

## 4. Discussion

GBS pigment is one of the major virulence factors and has been associated with cytolytic injury to mast cells [11], CD4+ T cells, and B cells [12], pyroptosis in human macrophages [13], bacterial penetration of the human placenta [7], and invasion of the amniotic cavity and fetal injury [14]. Furthermore, pro-inflammatory and pro-thrombotic activities have been reported, including blood clotting and accelerated plasma clotting [6]. Here, we show that GBS pigment initially activates human platelets before killing them via necrosis.

The pathomechanisms of pigment-mediated cell damage are still not fully understood. GBS predominantly colonize the gastrointestinal and vaginal tracts. It was shown that pigment promotes the invasion of placental cells by GBS, disrupts amniotic barriers, and enables the dissemination of bacteria [7]. Subsequently, pigment contributes to inflammation, neutrophil infiltration, and mast cell degranulation in infected tissues [15]. Pigment synthesis is encoded by the *cyl*-operon [7]. Another key virulence factor is the α2,3-linked sialic acid (Sia) capsule, which protects GBS from phagocytic killing [16]. Both factors are oppositely regulated via the CovR/S two-component system, and clinical isolates of hyper-hemolytic GBS (which harbor *covR/S* mutations) have reduced capsule levels [6,9,16]. We, and other researchers, have previously shown that: (i) The STSS/NF-HH and STSS/NF-LH variants exhibit low and high capsule expression, respectively. This phenomenon was exclusively linked to a deletion of three base pairs in the *covR* gene of STSS/NF-HH. (ii) Genes encoding for the pigment synthesis pathway are up-regulated in the LUMC16 strain to the same extent as in the STSS/NF-HH strain, although no mutations in *covR/S* were found [6,9]. (iii) GBS Sia blocks platelet activation [10]. On the basis of these findings, we hypothesized that reduced capsule levels and enhanced pigment expression will affect GBS–platelet interactions. Indeed, the infection of platelets with the pigmented strains resulted in dose- and time-dependent platelet activation, followed by a sequential loss of platelet viability. The activation phenotype was also observed after direct treatment with the pigment. In contrast, platelets retained the resting phenotype in infections with non-pigmented strains. These results indicate that GBS pigment is in fact the main driver of GBS-induced platelet interaction. However, the data also demonstrate that other GBS virulence factors contribute to platelet killing because GBS-mediated cytotoxicity towards platelets was more effective in bacterial infections compared to the application of the toxin alone. Our results also confirm previous observations that GBS strains resist platelet-induced killing via the capsule [10]. However, the aforementioned study utilized a GBS strain without hyper-hemolytic activity. Furthermore, caspase-3/7 activation was noted. Activated caspase-3 is a key executioner in apoptosis and requires upstream activation via a diverse range of cell death receptors and cleavage by the initiator caspase-8 [17,18]. However, although platelets express effectors of the extrinsic apoptotic pathway, no cell death receptors of the TNFR family, including Fas, have been identified so far [19,20,21]. Therefore, pigment-induced platelet killing could also occur due to caspase 3/7-dependent secondary necrosis [22]. Necrotic platelets display a procoagulant phenotype and are phosphatidylserine-positive on their surface. In addition, the loss of mitochondrial membrane potential, ballooning, and rupture of the cytoskeleton are described as features of platelet necrosis [23,24]. Our results show that pigmented GBS induce a reduction in platelet inner mitochondrial membrane potential. These results demonstrate that pigmented GBS induce necrosis. However, the stimulation of platelets with purified pigment had only a minor effect. Of note, a subpopulation of the buffer-control-treated platelets were also characterized by slightly reduced TMRE staining, although cell death was not detected in these control stimulations. Extraction of the GBS pigment requires high molecular weight stabilizers such as starch. Traces of starch remain within the purified fraction. Traces of starch potentially induce mitochondrial dysfunction, resulting in reduced mitochondrial membrane potential, however this is speculative. In line with this, it was shown that hyperglycemia could induce changes in mitochondrial morphology in otherwise healthy cells [25].

Overall, the expression of hemolytic and cytolytic toxins is not unique to GBS. Other Gram-positive bacteria, including *Streptococcus pneumoniae* (pneumolysin, Ply), *Staphylococcus aureus* (alpha-hemolysin, Hla) or group A streptococci (streptolysin O, SLO, and streptolysin S, SLS), express toxins with lytic activity. In contrast to GBS pigment, these toxins are proteins/peptides. Although the lysis of a variety of cell types, including platelets, is a common feature of these toxins [8], they act via different mechanisms [26,27] and are able to induce pyroptotic, apoptotic, and necrotic cell death [28]. Ply and SLO are cholesterol-dependent cytolysins. They form pores in cholesterol-rich membranes and use glycans as cellular receptors [28]. Furthermore, mannose receptor C-type 1 (CD206) was identified as a Ply receptor on human phagocytic cells [29]. This interaction results in the suppression of co-stimulatory molecules, leading to impaired maturation of phagocytes and subsequently abrogated T cell activation [29,30]. In contrast, SLS is a small non-immunogenic peptide that mainly targets erythrocytes, platelets, and leukocytes by a mechanism that is not yet fully understood [31]. Furthermore, Hla-induced cell damage is mediated through Hla-ADAM10 interactions on the host cell surface [32,33]. Similar to GBS pigment, Hla initially activates and then kills human platelets over time [34].

## 5. Conclusions

In conclusion, GBS pigment directly affects human platelets, leading to the initial activation and subsequent killing of platelets over time. However, only pigmented GBS, and not pigment alone, induced necrosis. It is possible that bacteria itself or cargo molecules are required to deliver the pigment into the intracellular environment. Further studies are needed in order to determine the specific events that lead to pigment-mediated platelet cell death.

## Data Availability

All data generated during this study are included in this article. Further enquiries can be directed to the corresponding authors upon reasonable request.

## Supplementary Materials

The following supporting information can be downloaded at: https://www.mdpi.com/article/10.3390/cells11101637/s1, Figure S1: Washed human platelets were infected with the pigmented LUMC16 and the non-pigmented LUMC16ΔcylX-K GBS strains at MOI 0.1, MOI 1.0, and MOI 10 or with the pigmented STSS/NF HH and the non-pigmented STSS/NF HH at MOI 0.1.; Figure S2: Washed human platelets were infected with the pigmented LUMC16 and the non-pigmented LUMC16ΔcylX-K GBS strains at MOI 0.1 or treated with increasing concentration of pigment and/or the respective buffer control.
