# Supplementary material for: Group B Streptococcal Hemolytic Pigment Impairs Platelet Function in a Two-Step Process
Source: Cells. 2022 May 13;11(10):1637. doi: 10.3390/cells11101637 (PMC9139542; doi:10.3390/cells11101637)
Supplement: Supplementary file 1 [file cells-11-01637-s001.zip › cells-1629645-supplementary.pdf]

Supplementary Material

Group B streptococcal hemolytic pigment impairs platelet function in a two-step process

Kristin Jahn [1], Patience Shumba [1], Phoenicia Quach [2], Mathias Müsken [3], Jan Wesche [4], Andreas Greinacher [4], Lakshmi Rajagopal [2, 5, 6], Sven Hammerschmidt [1*], and Nikolai Siemens [1*]

1 Department of Molecular Genetics and Infection Biology, Interfaculty Institute for Genetics and Functional Genomics, Center for Functional Genomics of Microbes, University of Greifswald, Germany; kristin.jahn@uni-greifswald.de (K.J.); patience.shumba@uni-greifswald.de (P.S.); sven.hammerschmidt@uni-greifswald.de (S.H.); nikolai.siemens@uni-greifswald.de (N.S.)
2 Department of Global Health, University of Washington, Seattle, Washington, USA; pjfquach@gmail.com (P.Q.); lakshmi.rajagopal@seattlechildrens.org (L.R.)
3 Central Facility for Microscopy, Helmholtz Centre for Infection Research, Braunschweig, Germany; mathias.muesken@helmholtz-hzi.de (M.M.)
4 Department of Transfusion Medicine, Institute of Immunology and Transfusion Medicine, University Medicine Greifswald, Germany; wesche@uni-greifswald.de (J.W.); andreas.greinacher@med.uni-greifswald.de (A.G.)
5 Center for Global Infectious Disease Research, Seattle Children's Research Institute, Seattle, Washington, USA
6 Department of Pediatrics, University of Washington School of Medicine, Seattle, Washington, USA
* Correspondence: nikolai.siemens@uni-greifswald.de; Tel.: +4938344205711 (N.S.); sven.hammerschmidt@uni-greifswald.de; Tel.: +4938344205701 (S.H.)

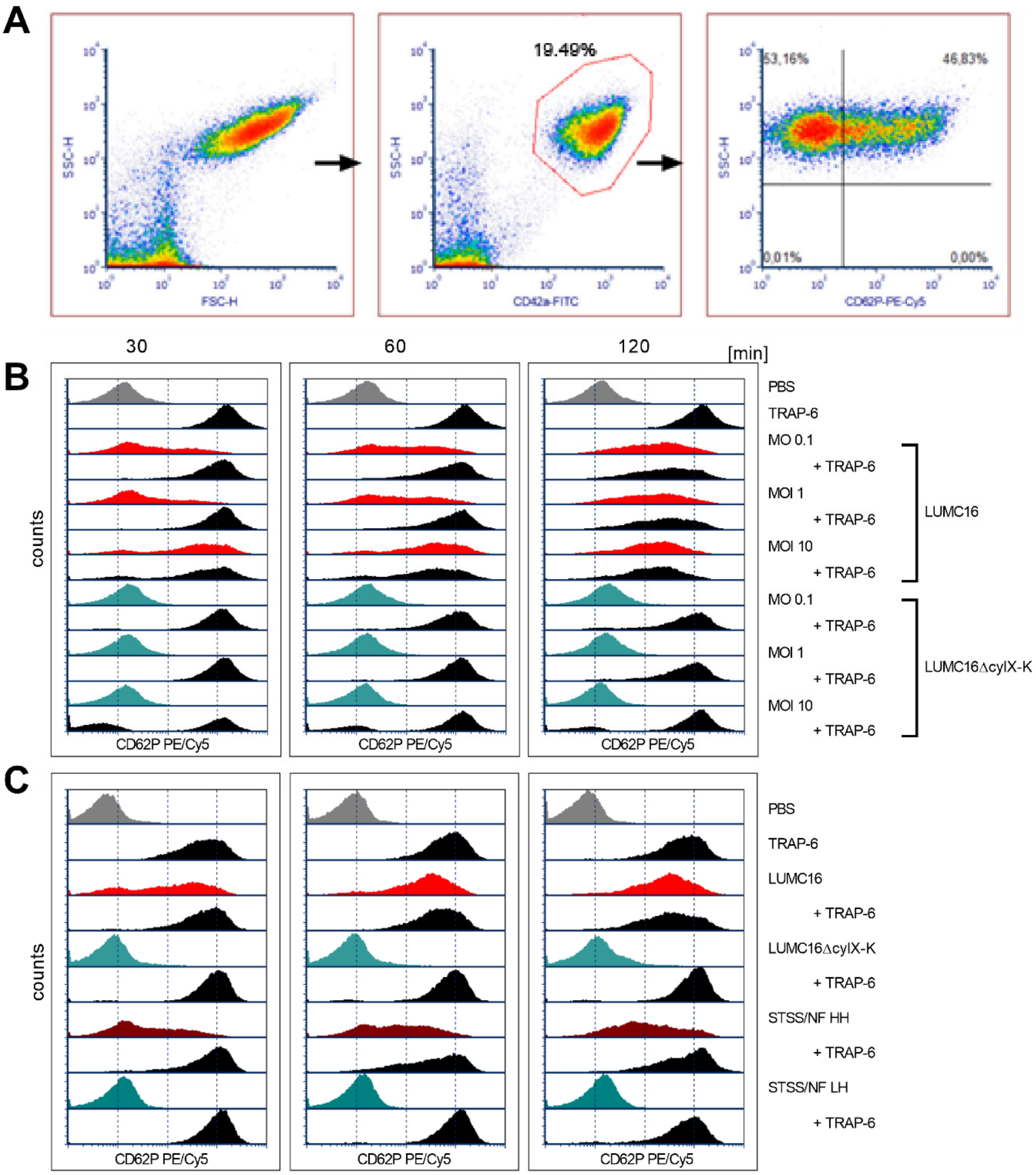

Figure S1. Washed human platelets were infected with the pigmented LUMC16 and the non-pigmented LUMC16Δ*cylX-K* GBS strains at MOI 0.1, MOI 1.0, and MOI 10 or with the pigmented STSS/NF HH and the non-pigmented STSS/NF HH at MOI 0.1. (A) Gating strategy of infected platelets. To exclude overlapping of bacteria and platelets in SSC/FSC scatter plot, platelets were labelled with CD42a. Activation of CD42a-positive cells was analyzed via CD62P PE/Cy5 staining. (B and C) Representative histograms for each platelet treatment/infection condition.

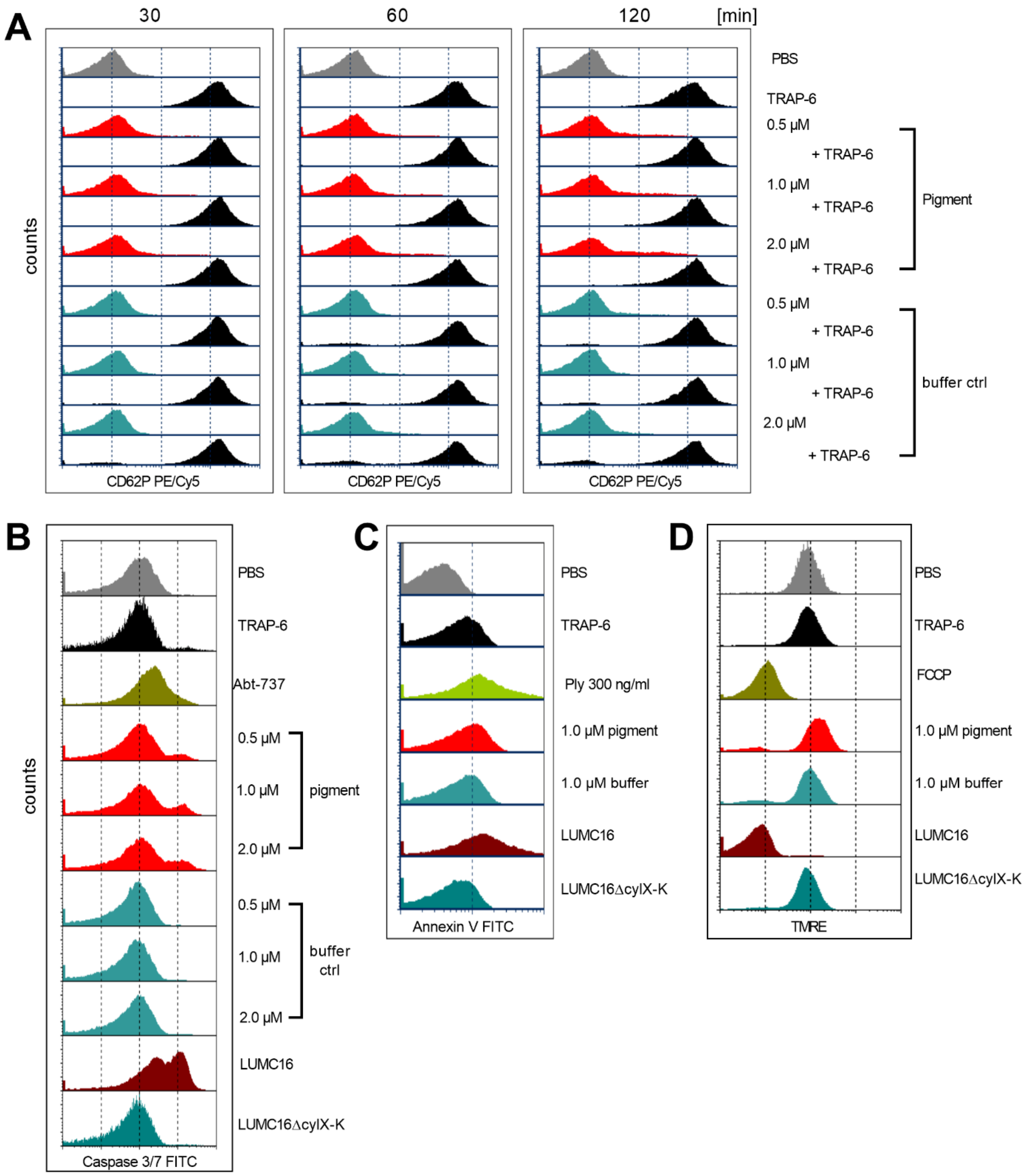

Figure S2. Washed human platelets were infected with the pigmented LUMC16 and the non-pigmented LUMC16Δ*cylX-K* GBS strains at MOI 0.1 or treated with increasing concentration of pigment and/or the respective buffer control. Shown are representative histograms for (A) CD62P dependent platelet activation, (B) caspase 3/7 activity, (C) phosphatidylserine positivity, and (D) changes of the inner mitochondrial membrane potential.
